# Solutions for the problems of silicon–carbon anode materials for lithium-ion batteries

**DOI:** 10.1098/rsos.172370

**Published:** 2018-06-06

**Authors:** Xuyan Liu, Xinjie Zhu, Deng Pan

**Affiliations:** School of Mechanical Engineering, University of Shanghai for Science and Technology, Shanghai 200093, People's Republic of China

**Keywords:** solutions, silicon–carbon, anode, lithium-ion batteries

## Abstract

Lithium-ion batteries are widely used in various industries, such as portable electronic devices, mobile phones, new energy car batteries, etc., and show great potential for more demanding applications like electric vehicles. Among advanced anode materials applied to lithium-ion batteries, silicon–carbon anodes have been explored extensively due to their high capacity, good operation potential, environmental friendliness and high abundance. Silicon–carbon anodes have demonstrated great potential as an anode material for lithium-ion batteries because they have perfectly improved the problems that existed in silicon anodes, such as the particle pulverization, shedding and failures of electrochemical performance during lithiation and delithiation. However, there are still some problems, such as low first discharge efficiency, poor conductivity and poor cycling performance, which need to be improved. This paper mainly presents some methods for solving the existing problems of silicon–carbon anode materials through different perspectives.

## 1. Introduction

With the development of social progress, increasing energy demands are becoming more urgent in various fields such as electronics, renewable energy generation systems and electric vehicles [1–4]. Lithium-ion batteries (LIBs) are considered as candidates for the increasing demand of portable electronic devices and electric and hybrid vehicles due to their high energy densities and stable cycle life. A secondary lithium-ion battery is fabricated with an anode, a cathode, a separator and electrolytes. Both the electrodes act as lithium ion hosts with a separator membrane to avoid a short circuit while the electrolyte supplies lithium ions. The specific energy of a battery is determined by the specific capacities of the cathode and anode materials [5]. Among various anode materials, silicon has attracted considerable attention because of its highest theoretical specific capacity (about 4200 mAh g^−1^), which is ten times higher than that of conventional carbon anodes (372 mAh g^−1^) and satisfactory potentials for lithium insertion and extraction (<0.5 V versus Li/Li^+^) [6].

Unfortunately, practical application of Si anodes is currently hampered by multiple challenges. The primary one is its huge volume change (approx. 300%) upon full lithiation and the resultant expansion/shrinkage stress during lithiation/delithiation, which induces severe cracking of Si. This results in the formation of an unstable solid electrolyte interphase (SEI) on the Si surface, and causes lithium trapping in active Si material, consequently leading to irreversible fast capacity loss and low initial coulombic efficiency (CE). Moreover, the slow lithium diffusion kinetics in Si (diffusion coefficient between 10^−14^ and 10^−13^ cm^2^ s^−1^) and low intrinsic electric conductivity of Si (10^−5^–10^−3^ S cm^−1^) also significantly affect the rate capability and full capacity utilization of Si electrodes [7–9]. Silicon nanostructure materials, including nanotubes, nanowires, nanorods, nanosheets, porous and hollow or encapsulating Si particles with protective coatings, have been devoted to achieve improved structural and electrical performance [10,11].

Meanwhile, the preparation methods for these nanostructures (e.g. vapour–liquid–solid methods, magnetron sputtering and chemical vapour deposition) have the disadvantages of complicated technologies and multiple steps [12,13]. Graphite and porous carbon are potential anode materials with relatively small volume change (e.g. graphite's volume expansion rate is about 10.6%) during the lithiation–delithiation process and have excellent cycle stability and electronic conductivity. Compared with silicon, carbon materials have a similar nature and they can combine closely with each other, so they are naturally selected as the substrate materials for dispersing silicon particles (i.e. dispersing carriers) [14,15]. Therefore, silicon–carbon composite anodes have been researched extensively because of their higher capacity, better electronic conductivity and cycle stability [16]. However, problems of silicon–carbon anode materials, such as low first discharge efficiency, poor conductivity and poor cycling performance need to be overcome. In this paper, we focus on the modification methods of silicon–carbon anode materials for LIBs. The status of solutions for the problems that exist with silicon–carbon anode materials is reviewed.

## 2. Preparation of the silicon–carbon materials

The Si–C anode materials are usually prepared by methods such as vapour deposition, high temperature solid phase synthesis, mechanical alloying, electrostatic electrospinning; the latter three methods require high temperature treatment. The methods mentioned above are the most widely used and easiest to implement.

### 2.1. Vapour deposition

Vapour deposition includes chemical vapour deposition (CVD) and physical vapour deposition (PVD). CVD is a chemical process used to produce high quality, high performance solid materials. The process is often used in the semiconductor industry to produce thin films. CVD is widely used in microfabrication processes to deposit materials in various forms, including monocrystalline, polycrystalline, amorphous and epitaxial. These materials include: silicon (SiO_2_, germanium, carbide, nitride and oxynitride), carbon (fibre, nanofibres, nanotubes, diamond and graphene), fluorocarbons, filaments, tungsten, titanium nitride and various high-k dielectrics. Chemical vapour deposition (CVD) in which hydrocarbons are decomposed over a substrate is perhaps the most popular route since it is a technique commonly adopted by the semiconductor industry and it is also relatively facile to set up in research laboratories [17]. PVD describes a variety of vacuum deposition methods, which can be used to produce thin films and coatings [18]. PVD is characterized by a process in which the material goes from a condensed phase to a vapour phase and then back to a thin film condensed phase. The most common PVD processes are sputtering and evaporation. PVD is applied in the manufacture of items that require thin films for mechanical, optical, chemical or electronic functions. Common industrial coatings applied by PVD are titanium nitride, zirconium nitride, chromium nitride and titanium aluminium nitride. Of the two vapour depositions, CVD is often used to prepare silicon–carbon composite materials [19].

### 2.2. High temperature solid phase synthesis

High temperature solid phase synthesis refers to a method that under high temperature (1000–1500°C) and through contact with a solid interface, reaction, nucleation and crystal growth response generates a large number of compound oxides. High temperature solid phase synthesis could be a common method to prepare Si/C composite materials, and in order to prevent the inert phase of Si/C the reaction temperature is often controlled under 1200°C [20].

### 2.3. Mechanical alloying

In contrast to high temperature solid phase synthesis, the materials prepared by mechanical alloying often have smaller particles, larger specific surface area and more uniform structures [21]. Mechanical alloying (MA) is a solid-state powder processing technique involving repeated cold welding, fracturing and re-welding of blended powder particles in a high-energy ball mill to produce a homogeneous material [22]. MA is now shown to be capable of synthesizing a variety of equilibrium and non-equilibrium alloy phases starting from blended elemental or pre-alloyed powders [23].

### 2.4. Electrostatic electrospinning

Electrospinning is a fibre production method, which uses electric force to draw charged threads of polymer solutions or polymer melts up to fibre diameters in the order of some hundred nanometres [24]. Electrospinning shares characteristics of both electrospraying and conventional solution dry spinning of fibres [25]. The process does not require the use of coagulation chemistry or high temperatures to produce solid threads from solution. This makes the process particularly suited to the production of fibres using large and complex molecules. Electrospinning from molten precursors is also practised and this method ensures that no solvent can be carried into the final products [26,27]. Electrospinning is a particularly low cost, simple and versatile method to produce nanofibres from various kinds of materials, and the improved coaxial electrospinning can fabricate nanotubes and core–shell structural nanofibres [28].

## 3. Modification of silicon–carbon anode materials

The main problems of silicon–carbon anode materials, such as low first discharge efficiency, poor conductivity and poor cycling performance need to be improved. When studying the modification of silicon–carbon anode materials, we usually take the following three aspects into consideration:

(1) Use different nanostructures to buffer the volume change of silicon, avoid the damage of generated SEI film on the electrode surface and avert the explosion of new surface during the process of circulation, in order to reduce the irreversible capacity loss and improve the cycling stability;
(2) We can significantly change the carbon material elements and the surface activity and improve the electrochemical properties through heteroatom doping, including non-metallic elements (boron, nitrogen, sulfur, phosphorus) and metal elements (K, Al, Ga, V, Ni, Co, Cu, Fe)
(3) Apply compound modification treatment by combining different forms of carbon with silicon to form uniform conductive network structures and to prepare silicon–carbon composite materials with good electrical conductivity, good adhesion and high chemical stability.

### 3.1. Structural modification of silicon–carbon anode materials

Carbon-based nanomaterials have unique properties that make them useful for many technical applications, including lightweight construction, electronics, energy generation, environmental technology and medicine [29–32]. Nanomaterials exhibit physical and chemical properties that are different from, and normally much better than, those of the bulk forms. These outstanding properties are often determined by the microstructure [33,34]. Carbon materials with excellent mechanical flexibility, high electronic conductivity and chemical stability in electrolytes have drawn much attention for the development of binder-free and lightweight electrodes [35]. The most recent advance in the applications of nanowires (NWs) [36,37], nanofibres (NFs) [38–41], nanotubes (NTs) [42–44] and nanospheres (NSs) [45–48] in the structure of silicon–carbon nanomaterials in LIBs are often mentioned. Table 1 lists some studies using combinations of Si–C anode materials (NWs, NFs, NSs), in the form of material structures and electrochemical properties. From these studies it can be seen clearly that anode materials with nanostructures can significantly improve the electrochemical cycling performance of lithium ion batteries.

**Table 1.** Electrical properties of silicon–carbon anodes with different structures. Q_r1_, the first reversible capacity; CE, coulombic efficiency; QdN(N), discharge capacity in Nth cycle; C.R.N., capacity retention in Nth cycle. NWs, nanowires; NFs, nanofibres; NTs, nanotubes; NSs, nanospheres.

anode material	structure	method	Q_r1_ (mAh g^−1^) (initial CE)	current density	QdN (mAh g^−1^) (N)	C.R.N %	ref.
C–SiNWs	NWs	CVD	1700.0	0.2 C	1300.0	76.5	[36]
			(90.0%)		(30)		
C–SiNWs	NWs	solution-based syntheses	2300.0	0.1 C	2000.0	87.0	[37]
			(96.0%)		(100)		
HCNFs–Si	NFs	CVD	941.4	0.6 C	733.9	77.9	[38]
			(78.5%)		(20)		
3D Si/C FP	NFs	electrospray/electrospinning technique	1589.0	0.5 A g^−1^	1267.0	79.7	[39]
			(80%)		(100)		
PC/Si NFs	NFs	electrospinning	1639.0	0.1 A g^−1^	1199.0	73.2	[40]
			(83.6%)		(10)		
Si–CNF–P	NFs	electrospinning	1957.0	2.0 A g^−1^	1187.0	60.6	[41]
			(79.3%)		(400)		
MWCNT@Si	NTs	magnesiothermic reduction	1547.0	0.4 A g^−1^	800.0	51.7	[42]
			(51.0%)		(10)		
MWCNT–Si	NTs	CVD	3000.0	0.3 A g^−1^	2280.0	76.0	[43]
			(96%)		(50)		
Si/ACNT	NTs	CVD	1496.0	0.1 A g^−1^	1198.0	80.0	[44]
			(66.4%)		(300)		
MSi@C	sphere	magnesiothermic reduction	1375.0	0.05 A g^−1^	1054.0	76.7	[16]
			(83.0%)		(100)		
Si@C NSs	sphere	chemical reduction	888.6	0.2 A g^−1^	610.7	68.7	[45]
			(52.0%)		(50)		
HSi@C	sphere	templating method and carbonization	1610.0	2.0 A g^−1^	800.0	49.7	[46]
			(70.0%)		(120)		
Si@void@C	sphere	CVD and magnesiothermic reduction	901.0	1.0 A g^−1^	796.0	88.3	[47]
			(62.5%)		(100)		
p-Si@C	sphere	partial magnesiothermic reduction	1287.0	0.5 A g^−1^	1146.0	89.1	[48]
			(69.4%)		(100)		

#### 3.1.1. Silicon–carbon nanowires

**Figure 1.**
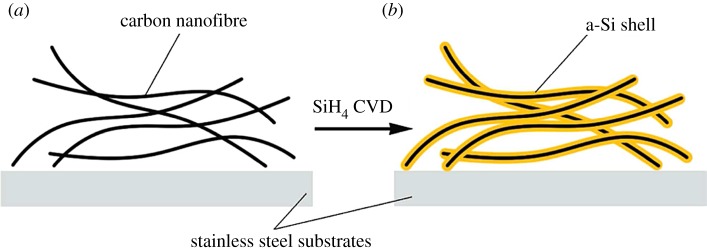
Schematic illustration of Si coating onto carbon nanofibres. (*a*) Bare CNFs. (*b*) C–Si core–shell NWs [36].

Nanowires are needed in many nanoscale applications. Various types of nanowires have been produced, including some with diameters ranging from about 50 to 100 nm [49]. The process of a novel design of carbon–silicon core–shell nanowires for high power and long life lithium-ion battery electrodes is schematically illustrated in figure 1 [36]. Amorphous silicon was coated onto carbon nanofibres to form a core–shell structure and the resulting core–shell nanowires showed great performance as anode material. They show a high charge storage capacity of about 2000 mAh g^−1^ and good cycling life. They also have a high coulombic efficiency of 90% for the first cycle and 98–99.6% for the following cycles. Bogart's group [37] reported a solution based synthesis of Si nanowires with a conductive carbon skin. Electrodes made with Si nanowires coated by pyrolysed carbon shells exhibited high capacities of over 2000 mAh g^−1^ for 100 cycles when cycled slowly at 0.1 C and over 1200 mAh g^−1^ when cycled quickly at 1.0 C. Uniform and complete carbon coatings were also found to prevent complete nanowire expansion needed for full lithiation of the nanowire.

#### 3.1.2. Silicon–carbon nanofibres

The attractive properties of catalytically grown carbon nanofibres (CNFs) have been known for decades [50]. They can be mass-produced inexpensively, and have excellent mechanical strength and high thermal and electrical conductivity [51]. The hybrid nanostructured Si/CNFs anodes exhibited superior device performance to that of materials used in previous studies, in terms of both specific capacity and cycle life. The CNFs provide not only a good strain/stress relaxation layer but also a conductive electron pathway [52].

Shu *et al*. [38] developed hollow carbon nanofibres/Si composites by a facile CVD technique with iron nitrate as the catalyst source and acetylene as the carbon source. They show excellent rate capability as anode materials for lithium batteries. The initial discharge and charge capacities of the CNFs/Si composites at 0.60 C are 1197.8 and 941.4 mAh g^−1^, respectively. A reversible charge capacity of 733.9 mAh g^−1^ can be delivered at 0.60 C after twenty cycles and the capacity retention is as high as 77.9%. It is found that CNFs/Si composites show superior electrochemical properties as anode materials for lithium batteries. They not only provide electronic conducting bridges between Si particles and the current collector for electron transportation but also act as a buffer to suppress the volume expansion of Si particles during lithiation and delithiation reactions.

**Figure 2.**
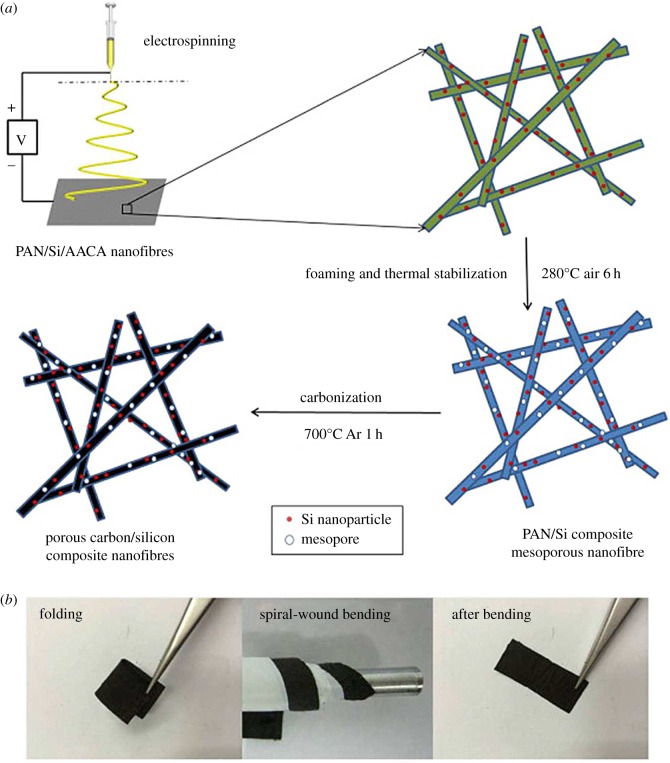
(*a*) Schematic of the electrospinning/foaming process in the manufacture of mesoporous C/Si/AACA composite nanofibres and (*b*) optical images of the C/Si/AACA composite nanofibres under mechanical deformation (folding and spiral-wound bending) and after bending [40]. Reproduced with permission from Wang *et al.* [40] (Copyright©2015 Elsevier).

**Figure 3.**
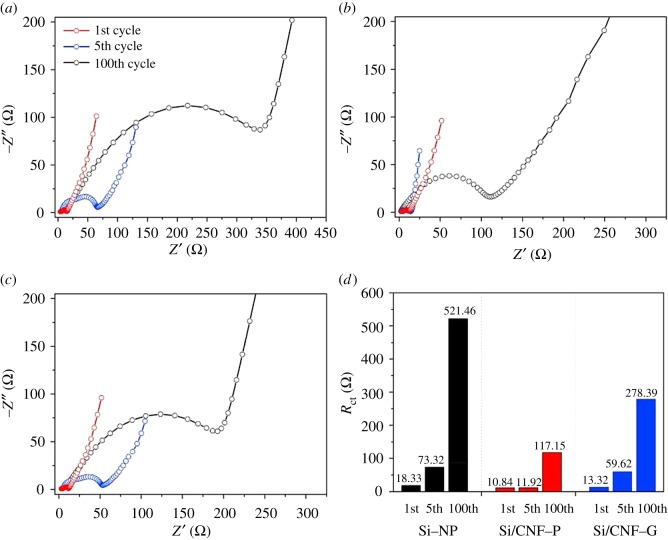
Nyquist plots of (*a*) Si–NP, (*b*) Si/CNF–P and (*c*) Si/CNF–G, and (*d*) the *R*_ct_ numbers measured after the 1st, 5th and 100th cycles [41]. Reproduced with permission from Kim *et al.* [41] (Copyright©2017 Elsevier).

A novel flexible three-dimensional (3-D) Si/C fibre paper electrode [39] is synthesized by simultaneously electrospraying nano-Si-PAN (polyacrylonitrile) clusters and electrospinning PAN fibres followed by carbonization. The flexible 3-D Si/C fibre paper electrode demonstrate a very high overall capacity of about 1600 mAh g^−1^ with capacity loss less than 0.079% per cycle for 600 cycles and excellent rate capability. Wang and co-workers [40] developed a new porous composite nanofibres manufacturing route, combining electrospinning and foaming processes (illustrated in figure 2). After 20 cycles ,when all these batteries reach stable charging/discharging rate, the discharging capacity shows as 1045 mAh g^−1^ for porous C/Si/AACA composite nanofibres. Kim *et al*. [41] introduced a 3-D paper-type Si–carbon nanofibre-composite electrode (Si/CNF–P) as a binder/current collector-free anode for LIBs that was prepared using an electrospinning method. Figure 3 shows Nyquist plots of the electrodes after the 1st, 5th and 100th cycles. Si–NP and Si/CNF–G exhibited a sharp deterioration in the discharge capacity, which might be due to a serious volumetric expansion by the alloy/dealloy process during the cycling. However, the Si/CNF–P exhibited a high initial capacity of 1957 mAh g^−1^ at 2 A g^−1^ and maintained 1187 mAh g^−1^ (retention rate of 60.6%) for 400 cycles. The enhanced cycling performance of Si/CNF–P might result from the suppression of the volumetric expansion of Si and facilitation of Li-ion transport. Si–NP and Si/CNF–G showed a severe increment of the charge transfer resistance (*R*_ct_) due to increased interface resistance after the cycling process, whereas Si/CNF–P showed a relatively slight increment of *R*_ct_ due to the stable electrode structure containing Si on CNF with voids, which could effectively release the volumetric expansion. These results confirm that the combination of CNFs and Si dramatically improves the electric conductivity and reduces the total cell resistance, which leads to the good performance of Si/CNF nanocomposite electrodes.

#### 3.1.3. Silicon–carbon nanotubes

Among all the conducting carbon materials, the carbon nanotube (CNT) is a one-dimensional material with a hollow tube including a curled graphene structure and an end cap with a hemispherical fullerene structure. CNTs, an allotrope of graphite, have been reported to show much improved lithium storage capacity compared with graphite, because of their unique structures and properties. CNTs have been reported to display conductivities as high as 106 S m^−1^ and 105 S m^−1^ for single-walled carbon nanotubes (SWCNTs) and multiwalled carbon nanotubes (MWCNTs), respectively. Recently, many investigations have focused on CNT-based anodes for LIBs with varying success, depending on the treatments employed [53]. Most previous reports on CNT-containing Si anode materials mainly emphasized the electrical connection of Si with the CNT exterior surface by simple mechanical mixing, growth of CNTs on Si, anchoring Si on the CNT surface, and deposition of Si on a CNT film to form a Si/CNT composite paper. However, the confinement effect of CNTs is not satisfactory due to the inhomogeneous distribution of Si particles and the fact that they are not strongly confined by the CNT network within a nanospace [54].

Nanostructures of Si nanobeads strung by CNTs [42–44] and Si nanotubes confined in CNTs [19] were proposed to accommodate huge volume changes of Si during lithiation and delithiation without appreciable mechanical failure. Chen's group [42] demonstrate the synthesis of uniform MWCNT@Si nanocomposites via the magnesiothermic reduction of pre-synthesized MWCNT@SiO_2_ nanocables [42]. The uniform MWCNT@Si nanocomposite electrode shows a capacity of approximately 900 mAh g^−1^ at 200 mA g^−1^. When the current density is reset to 400 mAh g^−1^, a capacity of 680 mAh g^−1^ can be retained, which indicates the good rate capability of the as-prepared uniform MWCNT@Si nanocomposite electrode.

**Figure 4.**
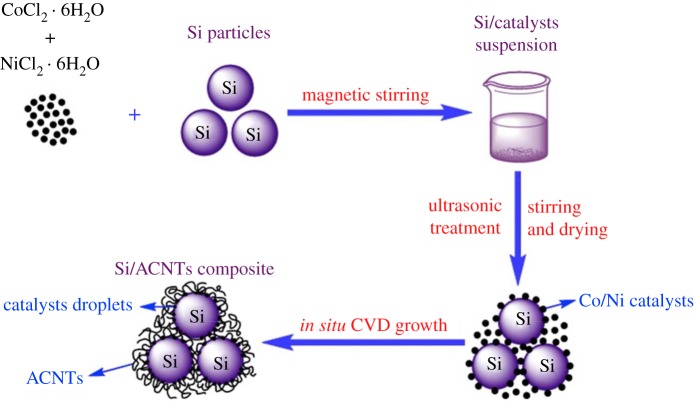
Schematic illustration presents the fabrication of Si/ACNT composite using impregnation and *in situ* CVD method to grow the coiled ACNTs onto the silicon particles [44].

**Figure 5.**
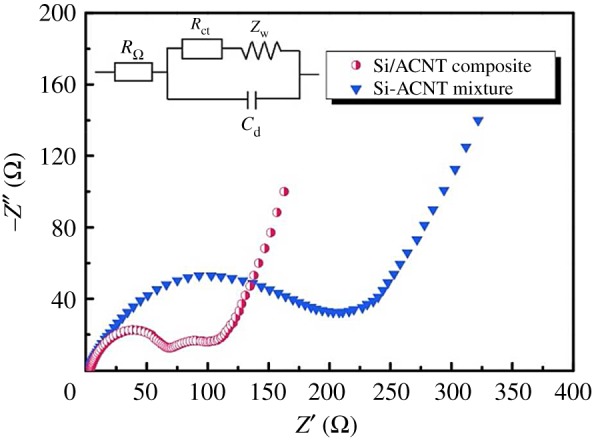
Nyquist plots of the cells with Si/ACNT composite and Si–ACNT mixture [44].

Epur *et al*. [43] reported a simple and facile, novel ascribable technique for achieving electrochemically active moderately thick Si–CNT nanocomposite coatings on copper foil. The Si–CNT heterostructures synthesized by the simple two-step CVD technique were compacted into a pellet using a conventional cold pressing technique that was then used to scribe the electrode on a copper foil to form the final electrode devoid of any additives and binders. A very high first discharge capacity of 3112 mAh g^−1^ was obtained followed by a low first cycle irreversible loss (19%). The scribed electrodes also exhibited good cyclability with 76% capacity retention at the end of 50 cycles, corresponding to a fade rate of 0.48% loss per cycle. A novel silicon core/amorphous carbon nanotube (ACNT) shell composite that can be used as LIB anode material was synthesized *in situ* by Zhao *et al.* [44] in the CVD growth process. The fabrication of Si/ACNT composite and Nyquist plots of the cells with Si/ACNT composite and Si–ACNT mixture are illustrated in figures 4 and 5, respectively. These curves unambiguously demonstrate that the Si/ACNT composite benefits from fast charge transfer, which can be reflected by the diameter of the semicircle in the high frequency region, indicating good electronic contact of Si/ACNT composite is maintained after repeated lithium insertion and extraction processes. It should benefit from the strong adherence and thus direct electronic connection of ACNTs on the surface of Si. This Si/ACNT composite delivered a high capacity of 1496 mAh g^−1^ at a current density of 100 mA g^−1^, and a superior cycling stability with 80% capacity retention after 300 cycles. This observed specific capacity improvement of Si/ACNT composite may be attributed to the formed 3-D conductive networks between silicon particles and interwoven ACNTs in the composite.

#### 3.1.4. Silicon–carbon nanospheres

Carbon nanospheres consist of graphite layers distributed discontinuously in the graphite structure with a state of glass phase [55]. Since the carbon nanospheres have high specific surface, excellent chemical stability and thermal stability, etc., they can be applied to preparation of high strength and high density C/C composite materials, high performance liquid chromatographic columns, high specific surface area activated carbon materials, LIB anode materials and a series of high-performance carbon materials. Carbon microspheres have a strong adsorption capacity and they can be used repeatedly [56,57].

**Figure 6.**
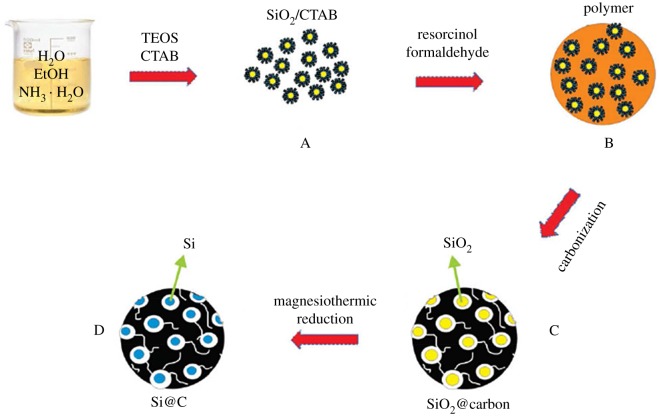
The schematic fabrication of mesoporous Si@C microspheres [16].

Hollow core–shell structured porous Si–C nanocomposites with void space were designed by Li's group [58] to accommodate the volume expansion during lithiation for high performance LIBs. An initial capacity of about 760 mAh g^−1^ after formation cycles (based on the entire electrode weight) with approximately 86% capacity retention over 100 cycles is achieved at a current density of 1.0 A g^−1^. Ma *et al*. [16] demonstrated the design and synthesis of novel mesoporous Si@C microspheres as anode materials for high-performance LIBs, which is illustrated in figure 6. They present a specific capacity of 1637 and 1375 mAh g^−1^ at first discharge and charge under a current density of 50 mA g^−1^. After 100 cycles, the charge capacity remains 1053 mAh g^−1^ with a coulombic efficiency of 99%, showing good cycle stability of the anode. Mesoporosity of Si@C microspheres effectively buffers the volume expansion/shrinkage of Si nanoparticles during Li ion insertion/extraction, which endows mesoporous Si@C microspheres with excellent electrochemical performance and cycle stability.

**Figure 7.**
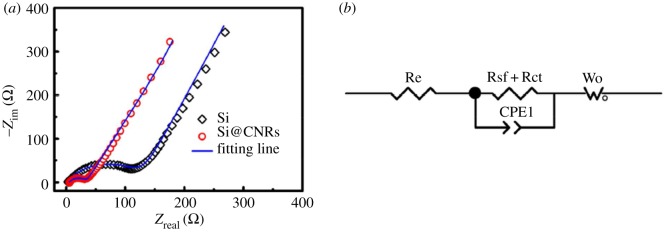
(*a*) Nyquist plots of Si and Si/carbon nanospheres composite; (*b*) equivalent circuit that is used to fit the EIS data [45]. Reproduced with permission from Zhou *et al*. [45] (Copyright © 2016 Elsevier).

**Figure 8.**
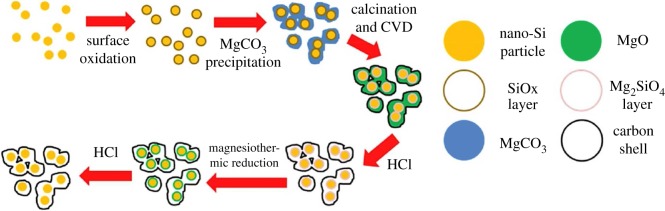
Schematic illustration of the synthesis of yolk–shell structured Si/C nanocomposite [47].

**Figure 9.**
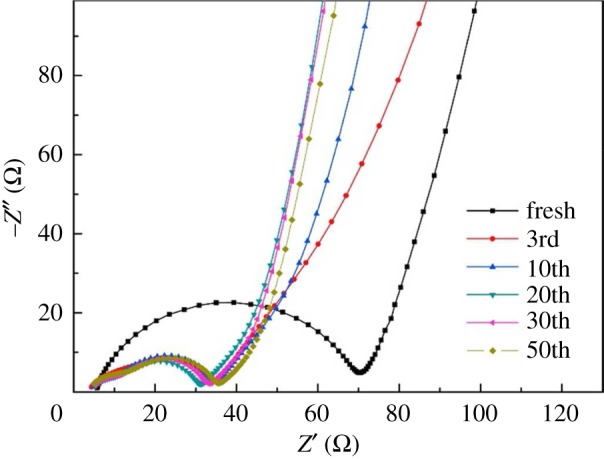
EIS of the SiVC-2 anode after different cycles [47].

Zhou *et al*. [45] prepared silicon/carbon nanospheres composite by a facile chemical method followed by heat treatment. The Si particles are coated with an amorphous carbon layer, which suppresses agglomeration of pristine Si. Carbon spheres accommodate large volume expansion of silicon during cycling. As shown in figure 7, the diameter of the semicircle for the Si/carbon nanospheres electrode is much smaller than that of the pure Si electrode, which indicates good electrical conductivity of silicon/carbon nanospheres composite. The silicon/carbon nanospheres composite exhibits initial reversible specific capacity of 888.6 mAh g^−1^ at current density of 200 mA g^−1^. After cycling up to 50 cycles, the electrode still delivers charge capacities of 610.7 mAh g^−1^. Ashuri *et al*. [46] have investigated the electrochemical properties of hollow silicon nanospheres encapsulated within a thin carbon shell, HSi@C, as a potential candidate for LIB anodes. The HSi@C nanospheres obtained deliver a stable specific capacity of 700 mAh g^−1^ after 100 cycles at a current density of 2 A g^−1^ and 800 mAh g^−1^ after 120 cycles at a current density of 1.0 A g^−1^. A yolk–shell structured Si-based anode was prepared by depositing MgO as sacrifice layer and CVD process, which is shown in figure 8 [47]. After 100 cycles at 1.0 A g^−1^, the Si@void@C electrode gave a specific capacity of 796 mAh g^−1^ with the capacity retention of 88.3%, which was higher than the Si@C produced directly by CVD. As is shown in figure 9, the electrochemical impedance spectroscopy (EIS) measurements indicate a good cycling stability and an improved electrical conductivity. The significant improvements for cycle performance demonstrate the yolk–shell structured Si–C nanocomposite in this work is a promising anode material for LIBs. A partial magnesiothermic reduction method, which is conducted by adjusting the proportion of added Mg powder to convert SiO_2_ into Si/SiO_2_ and subsequently to coat such a composite with a carbon layer was reported by Wu's group [48]. After removing unreacted SiO_2_ using HF, carbon-coated mesoporous Si (p-Si@C) materials can be obtained. The as-prepared p-Si@C shows superior electrochemical performance with a reversible capacity of 1146 mAh g^−1^ after 100 cycles at a rate of 0.5 A g^−1^.

### 3.2. Doping modification of silicon–carbon anode materials

Heteroatom doping can significantly change the carbon material elements, its surface activity and improve its electrochemical properties. Non-metallic elements (boron, nitrogen, sulfur, phosphorus) and metal elements (K, Al, Ga, V, Ni, Co, Cu, Fe) are often applied in doping modification of silicon–carbon materials. Among the heteroatoms, the atomic radius of the nitrogen atom is closer to that of the carbon atom than any other atoms, which makes it easier to replace carbon atoms in the atomic lattice of carbon materials to form the N-doped carbon material [59]. A nitrogen atom has one more extranuclear electron than a carbon atom, and with the very high electron affinity it can offer carbon atoms adjacent to the nitrogen atom a higher positive charge density. At the same time, there is a conjugate effect between lone pair electrons of nitrogen atoms and the big pi bond of the carbon atomic lattice. As a result, the nitrogen-doped carbon materials display excellent electrochemical properties and catalytic properties. Nowadays, the doping modification methods of silicon–carbon materials are mainly dependent on nitrogen doping.

#### 3.2.1. Si/nitrogen-doped carbon anode materials

The N-doped carbon layer with multiple type nitrogen is believed to deliver high electronic conductivity and electrochemical activity and help transport lithium ions in the interface due to defects caused by nitrogen doping [60–62]. Among heteroatoms for various doping sources, e.g. nitrogen or boron, nitrogen is the most attractive dopant in the carbon network, because the atomic size of nitrogen is comparable to that of carbon and its five valence electrons are available to form strong valence bonds with carbon atoms [63]. The nitrogen atoms incorporated into carbon networks should lead to the formation of stronger interactions between the nitrogen-doped carbon layer and lithium, which might be favourable for lithium insertion [64,65]. The N-doped layer could prevent direct contact between the electrode material and electrolyte. In addition, it is beneficial to improving the electronic conductivity of the composite and lithium ion transmission at the interface of the electrode and electrolyte [66]. Nitrogen-doped carbon-coating layers play a critical role in promoting and preserving the stable SEI layers and providing an efficient transport pathway for the electrons [67–69]. In table 2, we can see clearly that nitrogen-doped carbon materials significantly improve the conductivity and coulombic efficiency.

**Table 2.** Electrical properties of nitrogen-doped silicon–carbon composite anodes. Q_r1_, the first reversible capacity; CE, coulombic efficiency; QdN(N), discharge capacity in Nth cycle; C.R.N., capacity retention in Nth cycle. NC, nitrogen-doped carbon; SPs, spheres; RGO, reduced graphene oxide.

anode material	method	Q_r1_ (mAh g^−1^) (initial CE)	current density	QdN (mAh g^−1^) (N)	C.R.N (%)	ref.
CNCC–SPs	electrospray	1380.0	0.5 A g^−1^	1031.0	74.7	[70]
		(72.0%)		(100)		
NC@P–Si	combined approaches	2357.3	0.8 A g^−1^	1933.0	82.0	[71]
		(84.0%)		(100)		
Si@NC	ionic liquid assisted method	2602.0	0.42 A g^−1^	725.0	27.9	[72]
		(75.4%)		(100)		
Si–RGO/NCT	solution-mixing and carbonization process	2030.2	0.1 A g^−1^	892.3	44.0	[73]
		(76.2%)		(100)		
Si@NC NPs	laser photopyrolysis technique	769	1.0 C	697.5	90.7	[74]
		(95.0%)		(300)		
Si/P–NC	pre-template-coating and chemical acid etching	1846.3	1.0 A g^−1^	1730.0	93.7	[75]
		(99.0%)		(100)		

Zhang *et al*. [70] reported the fabrication of silicon/nitrogen-doped carbon/carbon nanotube (SNCC)nano/micro hierarchical structured spheres through a facile electrospray approach for the first time by using rice husk (RH) as silicon source. The unique hierarchical hybrid structure of the composite spheres contributes to fast electronic transport and prevents silicon from pulverization, possessing good structure stability upon the synergistic lithiation/delithiation of the components. These SNCC spheres could deliver a high reversible specific capacity of 1380 mAh g^−1^ at a current density of 0.5 A g^−1^, and still maintain 1031 mAh g^−1^ after 100 cycles.

An effective approach was developed to generate a nitrogen-doped carbon coating layer on porous silicon (CN@P–Si) to minimize the intrinsic drawbacks of low electrical conductivity and large volume expansion for LIBs [71]. The nitrogen-doped carbon coating layer shows more pronounced effect on the charge-transfer reaction resistance in the electrode–electrolyte interface. The cell with CN@P–Si electrode delivers a high specific capacity of 1904 mAh g^−1^ at the discharge current of 20 A g^−1^. After 100 cycles, the P-Si electrode with pores shows huge pulverization; in contrast the CN@P–Si electrode remains intact with reasonably low volume expansion.

**Figure 10.**
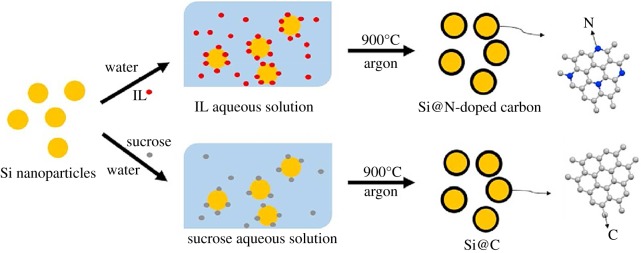
Schematic illustration of the synthesis process of Si@C and Si@N-doped carbon [72]. IL, ionic liquid.

**Figure 11.**
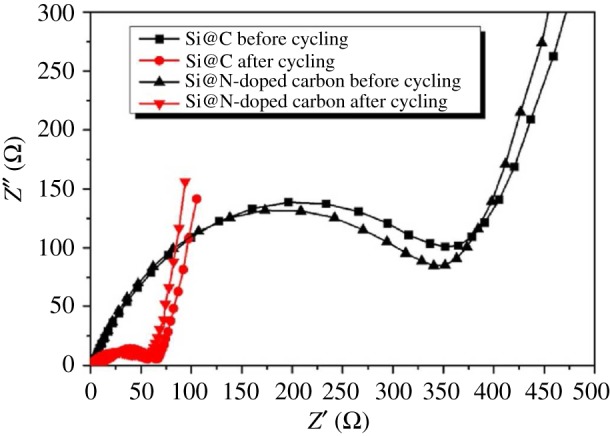
Impedance measurements for Si@N-doped carbon and Si@C before and after 100 cycles at 420 mA g^−1^ [72].

Shen's group [72] compared Si@N-doped carbon nanoparticles with Si@carbon nanoparticles, which is prepared by ionic liquid assist. The synthesis process of Si@C and Si@N-doped carbon is illustrated in figure 10. The as-prepared Si@N-doped carbon composite exhibited a high reversible capacity of 725 mAh g^−1^ after 100 discharge/charge cycles at a current density of 420 mAh g^−1^, about twice higher than that of Si@C(360 mAh g^−1^ after 100 cycles at 420 mA g^−1^). As shown in figure 11, the *R*_ct_ value of Si@N-doped carbon was smaller than that of Si@C before or after 100 cycles, which suggested a lower charge transfer resistance. In addition, the *Z*_w_ value of Si@N-doped carbon was larger than that of Si@C before or after 100 cycles, indicating a faster speed of Li-ion diffusion in the solid-state electrodes. These results demonstrated that compared with pure carbon, the N-doped carbon possesses faster Li^+^ ion diffusion and enhanced electronic conductivity. The improved electrochemical performance could be ascribed to the stable core–shell structure of the nanocomposite and more importantly the doping of nitrogen element into the carbon shell.

**Figure 12.**
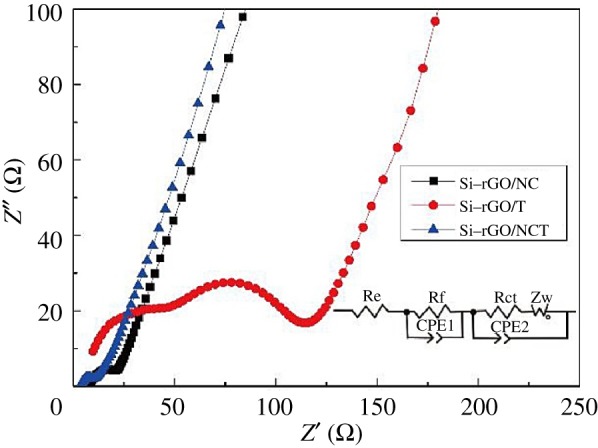
Nyquist plots of Si–rGO/NC, Si–rGO/T and Si–rGO/NCT composite electrodes after 10 cycles with amplitude of 5 mV in the frequency range from 100 kHz to 0.01 Hz, and the equivalent circuit used to model the impedance spectra (inset) [73].

**Figure 13.**
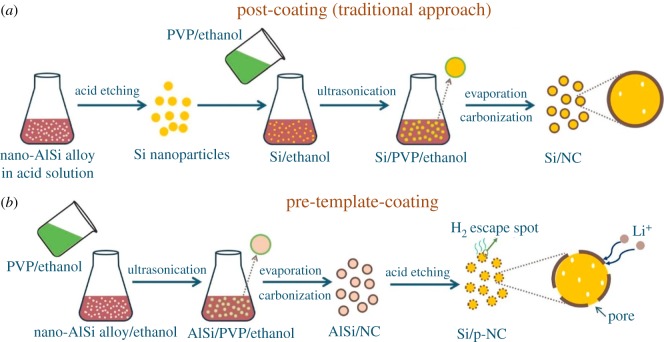
Schematic illustration of (*a*) the post-coating approach for Si/NC and (*b*) the pre-template-coating approach for Si/p-NC from a low-cost Al-Si alloy powder [75].

A Si–rGO/NCT composite was synthesized by Tang *et al*. [73], in which Si nanoparticles (SiNPs) are enwrapped with N-doped carbon and combine with N-doped graphene and CNTs as conductive matrices. As is obvious in figure 12, the Si–rGO/NCT electrode has the smallest semicircle diameter compared with the Si-rGO/NC electrode and Si–rGO/T electrode, indicating the lowest charge transfer resistance during the electrochemical reaction. The EIS result indicates that superior cycle and rate performances of Si–rGO/NCT are achieved with the well-protected SiNPs and excellent conductivity provided by the N-doping carbon and CNTs. The Si–rGO/NCT composite exhibits high specific capacity and good cycling stability (892.3 mAh g^−1^ at 100 mA g^−1^ up to 100 cycles), as well as improved rate capability. The N-doped carbon outside SiNPs can not only improve the electrical conductivity of the composite, but also buffer the stress caused by huge volume change of SiNPs during the lithiation/delithiation process. Choi *et al*. [74] fabricated Si@NC NPs using pyrrole and FeCl_3_, and the coating layer thickness was controlled by varying the amount of added FeCl_3_. The Si@NC NP sample with 1 g of FeCl_3_ showed a specific capacity of 967 mAh g^−1^, and that with 1.5 g of FeCl_3_ retained 90.7% of its initial capacity (769 mAh g^−1^); this is quite a high value compared with that of Si (38.3%). The enhanced cycling stability of the Si@NC NPs was attributed to the formation of C–N networks resulting from the presence of polypyrrole. A facile approach was presented by Zhou's group [75] for synthesizing silicon/porous nitrogen-doped carbon composite with a unique core–porous shell structure via pre-template-coating and chemical acid etching methods. In figure 13, the traditional post-coating approach for Si/NC and the pre-template-coating approach for Si/p-NC are illustrated. The silicon/porous nitrogen-doped carbon composite with 88% Si delivers a high reversible capacity of 1730 mAh g^−1^ (based on the total mass of the composite) after 100 cycles at a current density of 1000 mA g^−1^ with a coulombic efficiency of approximately 100%. Moreover, a long cycle life at a high rate is also achieved, with a notable capacity of 665 mAh g^−1^ after 600 cycles at a high current density of 5000 mA g^−1^.

### 3.3. Compound modification of silicon–carbon anode materials

Within the mixed silicon and carbon anode materials, silicon and carbon are closely combined to form a stable and uniform system. In the process of charging and discharging, silicon is the active centre of electrochemical reaction and the carbon carrier has the effect of lithiation and delithiation. Besides, the carbon carrier can also be an electron transport channel and structural support. Through making compound modification by combining different series of carbon materials with silicon, composite materials with uniform conductive network structures, good electrical conductivity, good adhesion and high chemical stability can be prepared. From table 3, we can clearly see that the silicon–carbon composite anode materials with different components can significantly improve the electrochemical cycling performance of LIBs.

**Table 3.** Electrical properties of silicon–carbon composite anodes with different components. Q_r1_, the first reversible capacity; CE, coulombic efficiency; QdN(N), discharge capacity in Nth cycle; C.R.N., capacity retention in Nth cycle. G, graphite; Gr, graphene; RGO, reduced graphene oxide.

anode material	method	Q_r1_ (mAh g^−1^) (initial CE)	current density	QdN (mAh g^−1^) (N)	C.R.N (%)	ref.
G/p-Si/AC	spray drying/pyrolysis synthesis	723.8	0.1 A g^−1^	592.4	81.8	[76]
		(75.2%)		(100)		
G/Si@C	mechanical milling, spray drying, pitch coating and pyrolysis	818.8	0.1 C	637.7	89.5	[77]
		(77.9%)		(100)		
HC–nSi/G	hydrothermal carbonization	1071.6	0.5 C	878.6	81.8	[78]
		(80.5)		(100)		
Si/C@NGs	spray-drying-assisted self-assembly method	483.3	0.1 A g^−1^	428.1	88.6	[79]
		(82.8%)		(100)		
AG/PNSi@C	spray drying	553.0	0.1 A g^−1^	449.4	81.3	[80]
		(81.0%)		(500)		
p-Si/G/CNTs@C	spray drying	863.2	0.1 A g^−1^	701.8	81.3	[81]
		(81.6%)		(100)		
Si@C/G	high energy wet ball-milling and pyrolysis	786.0	0.2 A g^−1^	645.0	82.1	[82]
		(62.4%)		(300)		
Si/CNFs@RGO	electrostatic self-assembly method and hydrothermal dehydration	2608.4	0.1 A g^−1^	1055.1	40.5	[83]
		(73.2%)		(130)		
p-Si/C/RGO	spray drying and carbonization	945.0	0.1 A g^−1^	928.0	98.0	[84]
		(65%)		(70)		
Si–CNTs/G paper	acid etching	1200.0	0.2 A g^−1^	1100.0	91.7	[85]
		(57.1%)		(100)		
Si@RGO@CNFs	electrospinning	1228.0	0.8 A g^−1^	887.0	72.0	[86]
		(71.5%)		(100)		
sm-Si@C/Gr	hydrothermal assembly	1423.0	1.0 A g^−1^	1192.0	84.0	[87]
		(71.5%)		(100)		
Si@C@RGO	spray drying and calcination	1599	0.2 A g^−1^	1517.5	94.9	[88]
		(75.3%)		(100)		

#### 3.3.1. Si/carbon/graphite anode materials

The biggest problem for using silicon as anode is the huge volumetric expansion up to 300% when silicon is maximally lithiated [89,90]. One of the ways to reduce the large volumetric expansion effect and make great use of the large specific capacity of silicon is blending graphite and silicon [91]. Graphite is a good candidate for a new anode material because it has great advantages of stability and cheaper cost as well as low working voltage [92]. We may use a composite of graphite, carbon and silicon to provide affordable anode capacity with minimized anode volume expansion [93,94].

A silicon/graphite/amorphous carbon (Si/C) composite with a low silicon content in a core–shell structure was synthesized by the spray drying method [76]. The combination of the core–shell structure for the composite and a porous carbon-coating layer accommodates the large volume change of the silicon during the lithium intercalation/extraction process, thus stabilizing the electrode structure during discharge/charge cycles. The as-obtained Si/C composite demonstrates high capacity and excellent cycle stability with an initial specific discharge capacity of approximately 723.8 mAh g^−1^ and a reversible specific capacity of approximately 600 mAh g^−1^ after 100 cycles at a constant density of 100 mA g^−1^.

**Figure 14.**
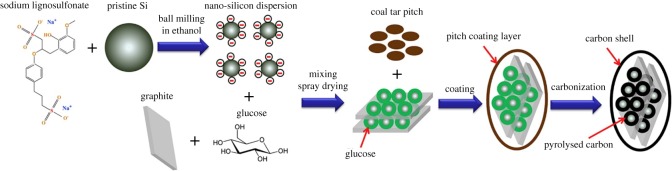
Schematic illustration of the synthesis procedures of G/Si@C [77].

Li *et al*. [77] developed a scalable and cost-effective method, including the processes of mechanical milling, spray drying, pitch coating and pyrolysis, to fabricate a core–shell structured graphite/silicon@pyrolysed-carbon (G/Si@C) composite. The synthesis procedures of G/Si@C is illustrated in figure 14. As a negative electrode material of LIBs, the synthesized G/Si@C composite has excellent structural stability and electrochemical performance. The G/Si@C composite with 15.7 wt% silicon shows a high reversible capacity of 637.7 mAh g^−1^ with an initial efficiency of 77.9%, the capacity retention is 89.5% after 100 cycles. Jeong's group [78] demonstrated a cost-effective hydrothermal carbonization approach to prepare a hard carbon-coated nano-Si/graphite (HC–nSi/G) composite as a high performance anode for LIBs [78]. In this hierarchical structured composite, the hard carbon coating not only provides an efficient pathway for electron transfer, but also alleviates the volume variation of Si during charge/discharge processes. The HC–nSi/G composite electrode shows excellent performance, including a high specific capacity of 878.6 mAh g^−1^ based on the total weight of composite, good rate performance and a decent cycling stability, which is promising for practical applications.

**Figure 15.**
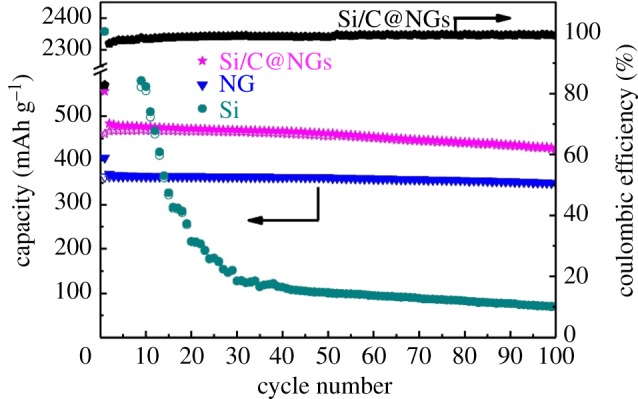
The cycling performance of Si/C@NGs, NG and Si for 100 cycles between 0.01 and 1.5 V at a current density of 0.1 A g^−1^ [79].

A type of Si/C@NGs composite containing flake-shaped sub-micron sized silicon (Si) enwrapped by pyrolysed carbon and natural graphite (NG) was successfully prepared by Wang's group [79] via a spray-drying-assisted self-assembly method. The Si/C@NGs composite with hierarchical structure was produced by the granulation of natural graphite (NG) particles and SAN/Si composite microspheres via spray drying and pyrolysis. Compared with pure silicon and natural graphite, which is shown in figure 15, the as-synthesized Si/C@NGs composite exhibits better performance with an initial efficiency of 82.8% and a capacity retention of 428.1 mAh g^−1^ (1524.0 mAh g^−1^ versus Si) after 100 cycles at 0.1 A g^−1^. The better cycling stability of Si/C@NGs than that of pure Si and higher capacity than that of the NG electrode could be attributed to the reasonable loading of Si (6.7 wt%) and rational structural design by applying SAN and graphite backbones. Chen *et al*. [80] have prepared a carbon-coated core–shell structure artificial graphite@plasma nanosilicon@carbon (AG@PNSi@C) composite as LIB anode material via a spray drying method. The as-prepared composite shows superior performance as anode in LIBs with a discharge capacity of 553 mAh g^−1^ and a recharge capacity of 448 mAh g^−1^. Besides the remarkable electrochemical performances, the facile and mass-producible synthesis process makes the AG@PNSi@C composite very promising for its application in LIBs. A porous Si-based composite has been reported, which consists of nano silicon (obtaining high capacity), graphite (gaining stable structure), carbon nanotube (increasing electron conductivity), and pitch (porous structure as well as a binder), prepared by a spray-drying method [81]. It shows an initial reversible capacity of 863.2 mAh g^−1^ at 100 mA g^−1^, and exhibits capacity retention of 81.3% after 100 cycles. The composite also possesses good rate capability, and up to 89.3% of the reversible capacity can be recovered at 1.0 A g^−1^. Graphite ensures the structural stability of the composite and help the dispersion of Si particles.

#### 3.3.2. Si/carbon/graphene anode materials

The isolation of a single layer of graphite, known today as graphene, not only demonstrated amazing new properties but also paved the way for a new class of materials often referred to as two-dimensional (2-D) materials [95]. Graphene has either planar or 3-D morphology [96–101]. Graphene in 2-D planar form has been intensively explored in fundamental physics and surface chemistry [102–105], electronics [106,107] and (*b*) optoelectronics [108,109], but it is less studied in energy [110–112], environmental [113,114] and biomedical applications [115]. Three-dimensional nanostructured graphene can be used as a replacement or enrichment material [116]. One of the more common routes to fabricate graphene is by CVD, which has emerged as the dominant synthesis route since it is already a well-established process in both industry and laboratories [117–119]. In recent years, graphene has been proposed as one of the best active carbon sources to prepare silicon-based composite anodes due to its excellent properties such as high conductivity, high mechanical strength, high chemical stability, super-high specific surface area and open porous structure. Graphene plays a flexible confinement function for tolerating volume changes to the composite in LIBs [120–122]. Since graphene has large surface area, high electrical conductivity and discharge capacity, it is an attractive carbon material to improve the electrochemical performance of the Si-based composite electrodes, leading to a more enhanced cycle ability at large current densities [123–125].

**Figure 16.**
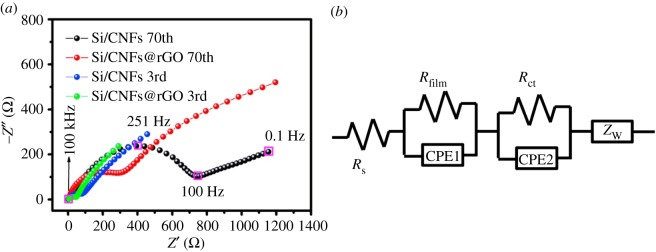
(*a*) Nyquist plots of Si/CNFs and Si/CNFs@rGO electrodes after different cycle numbers from 100 kHz to 0.01 Hz in the fully charged state and (*b*) corresponding equivalent circuit for the system [83]. Reproduced with permission from Chen *et al*. [83] (Copyright © 2016 Elsevier).

**Figure 17.**
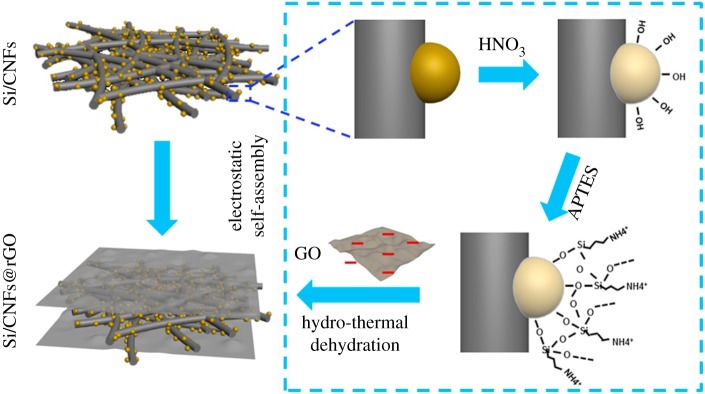
Schematic fabrication process of the sandwiched Si/CNFs@rGO composite [83].

Sun *et al.* [82] developed a novel approach to prepare silicon@carbon/graphene sheets (Si@C/G) composite with a unique structure, in which carbon coated Si nanoparticles are uniformly dispersed in a matrix of graphene sheets, to enhance the cycle ability and electronic conductivity of Si-based anodes for Li-ion batteries. It exhibits a high Li-storage capacity of 1259 mAh g^−1^ at a current density of 0.2 A g^−1^ in the first cycle. Further, a stable cycle ability with 99.1/88.2% capacity retention from initial reversible charge capacity can be achieved over 100/300 cycles, showing great promise for battery applications. Chen *et al.* [83] prepared a sandwich-structured silicon-based anode prepared to inhibit the fragmentation of silicon electrodes typically caused by the large volume changes that occur during charge/discharge processes. The Si/CNFs@rGO composite exhibits a high specific capacity of 1055.1 mAh g^−1^ up to 130 cycles at 0.1 A g^−1^, with slight capacity loss. The Si/CNFs@rGO electrode also demonstrates outstanding rate behaviour with a reversible capacity of 358.2 mAh g^−1^ at 5.0 A g^−1^. In figure 16, the electrical conductivities of the as-prepared Si/CNFs and Si/CNFs@rGO, performed by a DC technique, indicates the enhanced electrical conductivity by introduction of the protecting layer. That is, the reduced graphene layer significantly improves the electrical conductivity and structural integrity of the electrode. As is illustrated in figure 17, an electrostatic self-assembly method and hydrothermal dehydration are used to introduce a reduced graphene oxide layer (rGO) on the surface of silicon/carbon nanofibres (Si/CNFs), which prevents the exfoliation of nano-Si from the electrode bulk to the liquid electrolyte, reduces the electric contact loss, stabilizes the electrode's structural integrity and improves electrochemical conductivity.

**Figure 18.**
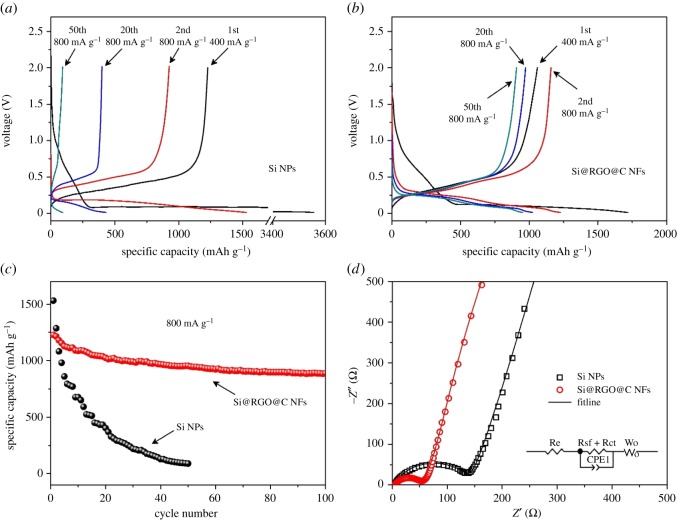
Typical charge/discharge curves of (*a*) Si NPs and (*b*) Si@RGO@C NFs; (*c*) cycle properties of Si NPs and Si@RGO@C NFs; (*d*) impedance spectra of Si NPs and Si@RGO@CNFs, the inset figure shows the equivalent circuit for the plot fitting [86].

Tao's group [84] have prepared porous Si/C/reduced graphene oxide (Si/C/rGO) microspheres by the spray drying and subsequent carbonization process using polyvinyl alcohol (PVA) as cross-linking agent. The designed micrometre-size reduced graphene oxide wrapped Si/C ball structure offers a buffer space for the volume change of Si during the charge–discharge process. The fabricated Si/C/rGO microspheres exhibit a high reversible capacity of 928 mAh g^−1^ after 70 cycles at a current density of 100 mA g^−1^, good rate capability and cycling stability. A flexible self-standing Si–CNT/graphene paper was fabricated with 3-D sandwich-like structure by Cai's group [85] after combining with graphene sheets. The self-standing Si–CNT/graphene paper anode exhibited a high specific capacity of 1100 mAh g^−1^ even after 100 cycles at 200 mA g^−1^ current density with a coulombic efficiency of above 99%. The silicon/graphene/carbon composite nanofibres (Si@RGO@C NFs) with a hierarchical structure were prepared by encapsulating graphene-coated Si nanoparticles in the interconnected carbon nanofibres based on electrospinning technology [86]. As is exhibited in figure 18, the well-defined Si@RGO@C NFs demonstrate a better electrochemical performance with a reversible capacity of 1228 mAh g^−1^ and a capacity retention of 72% after 100 cycles with a current density of 800 mA g^−1^. With the current density gradually increasing to 4000 mA g^−1^, the electrode displays a specific capacity of 954 mAh g^−1^, exhibiting superior rate capability compared to the Si nanoparticles. These excellent electrochemical properties are attributed to the hierarchical core–shell structure and cross-linked network for Si@RGO@C NFs.

**Figure 19.**
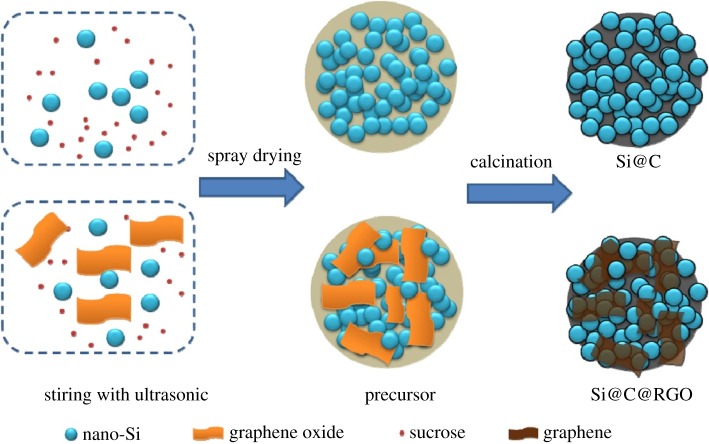
Schematic illustration of the synthesis of Si@C@RGO [88].

**Figure 20.**
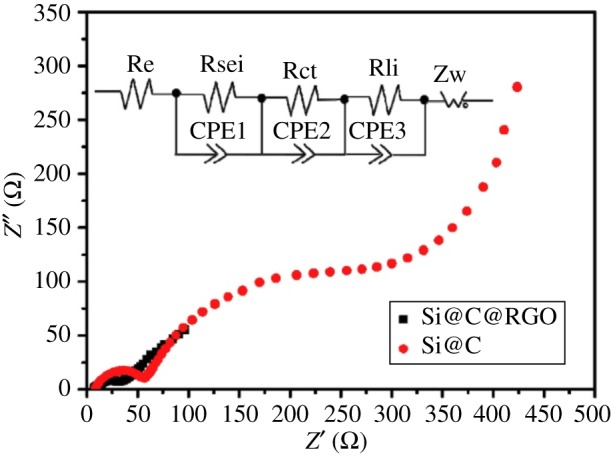
Nyquist plots of the Si@C@RGO and Si@C composite anodes [88].

Lee *et al*. [87] provided a new opportunity based on Si waste in fabricating sustainable and scalable Si-based anodes for high-capacity LIBs. During the electrode fabrication, the sub-micron Si particles were encapsulated with 3-D carbon matrix including a carbon coating on the Si particles and interconnected reduced graphene layers, which can effectively mitigate volume variation of the Si as well as supporting electrical conductivity. The sub-micron Si particle-based electrodes exhibit a reversible capacity of 1192 mAh g^−1^ at 100th cycle, retaining up to 84% of initial capacity. Pan *et al*. [88] have prepared a micro-sized silicon@carbon@graphene spherical composite (Si@C@RGO) by an industrially scalable spray drying approach and a subsequent calcination process. The obtained Si@C@RGO anode exhibits a high initial reversible specific capacity of 1599 mAh g^−1^ at a current density of 100 mA g^−1^ with a good capacity retention of 94.9% of the original charge capacity at a higher current density of 200 mA g^−1^. Moreover, the Si@C@RGO anode shows a high reversible specific capacity of 951 mAh g^−1^ even at a high current density of 2000 mA g^−1^. As is illustrated in figure 19, the combination of carbon shells and flexible graphene can effectively enhance the electrical conductivity of the composite and accommodate significant volume changes of silicon during cycling. According to the fitting results (based on equivalent circuit in figure 20), we find that the incorporation of graphene can inhibit the growth of the SEI layer and the unique structure of the Si@C@RGO composite can effectively enhance the conductivity and increase the cycling stability of the electrode.

From the literature survey above, we can conclude that graphene is an effective buffer element to prevent structural changes of silicon against volume expansion and extraction of the electrode during Li alloying/de-alloying processes because graphene greatly improves the reversible capacity, cycling stability and rate capability [126,127].

## 4. Conclusion and perspective

In general, the research on silicon–carbon anode materials is mainly aimed at the development of a higher energy density, greater charge–discharge performance, stable cycle performance and higher safety performance aspects, and the development of large-scale preparations of low cost, stable performance silicon–carbon composite materials. Methods such as structural modification can effectively increase the surface area, thus the first reversible capacity of the silicon–carbon anode material is improved. Heteroatomic doping can change the conductivity of the material and has effectively improved the coulomb efficiency of silicon–carbon anode materials. Through combination with carbon materials with excellent mechanical flexibility, high electronic conductivity and chemical stability in the electrolyte, we can clearly find that the cycling stability of silicon–carbon anode materials is greatly improved. In addition, research on the mechanism of lithiation–delithiation and the exploration of adhesives and electrolytes that are more compatible with the silicon–carbon materials will also be hot topics in the following 50 years.

## Data accessibility

This article has no additional data.
